# Non-motor Behavioral Alterations of PGC-1α-Deficient Mice – A Peculiar Phenotype With Slight Male Preponderance and No Apparent Progression

**DOI:** 10.3389/fnbeh.2018.00180

**Published:** 2018-08-27

**Authors:** Levente Szalardy, Mate F. Molnar, Denes Zadori, Edina K. Cseh, Gabor Veres, Gabor G. Kovacs, Laszlo Vecsei, Peter Klivenyi

**Affiliations:** ^1^Department of Neurology, Faculty of Medicine, Albert Szent-Györgyi Clinical Center, University of Szeged, Szeged, Hungary; ^2^MTA-SZTE Neuroscience Research Group, Szeged, Hungary; ^3^Institute of Neurology, Medical University of Vienna, Vienna, Austria

**Keywords:** anxiety, behavior, depression, memory, peroxisome proliferator-activated receptor gamma coactivator, PGC-1α

## Abstract

Dysfunction of peroxisome proliferator-activated receptor gamma coactivator-1alpha (PGC-1α) has been linked to various neurodegenerative and neuropsychiatric disorders; however, reports on psychic behavioral alterations on PGC-1α-deficient animals are sparse. The present study revisited prior observations of anxiety-related, depression-related, and hippocampal memory-related observations having been made on different PGC-1α-deficient murine strains, in a large-scale analysis on whole-body full-length (FL-)PGC-1α-deficient mice. The examinations were performed on animals covering a wide age range enrolled from both sexes, and included paradigms such as the open-field, elevated plus maze, light-dark box, tail suspension test, and spatial recognition two-trial Y-maze. The findings revealed no signs of previously reported anxiety-like behavior, but revealed an unexpected phenotype with decreased anxiety behavior consistent throughout different paradigms, with slight male preponderance. This was associated with despair-like anhedonic behavior, consistent with that reported previously, but did not associate with either peripheral or brain alterations in kynurenic acid synthesis, which was previously proposed. Though male FL-PGC-1α-deficient mice tended to perform poorer in the hippocampus-based spatial learning paradigm, the genotype overall was not associated with impairment in spatial memory, contradicting with prior observations. None of the observed alterations deteriorated with age, similarly to motor alterations as reported previously. The most likely contributors of this peculiar phenotype are discussed, with clinicopathological correlations drawn. Being the first to address these behavioral domains within the same PGC-1α-deficient strain, our findings extend the knowledge about the complex *in vivo* effect of PGC-1α dysfunction and add important notes to research in the field of PGC-1α in connection with neuropsychiatric disorders.

## Introduction

Peroxisome proliferator-activated receptor gamma (PPARγ) coactivator-1alpha (PGC-1α), a widely studied metabolic master regulator protein, is responsible for the coactivation of a number of nuclear transcriptional factors that in turn enhance the expression of several genes involved in the promotion of mitochondrial adaptive biogenesis, oxidative phosphorylation, and oxidative stress defense (Szalardy et al., 2015). In addition to its role in diseases related to fat and glucose metabolism (Wu et al., 2016), the deficient function of PGC-1α has been implicated in the pathogenesis of various CNS diseases, especially where defective mitochondrial function has been described as one of the key pathogenic factors, such as in HD, AD, PD, and MND (Rona-Voros and Weydt, 2010; Szalardy et al., 2015). Furthermore, increasing evidence indicate a potential role of PGC-1α in various psychiatric disorders, including schizophrenia (McMeekin et al., 2016) and depression (Agudelo et al., 2014). Several studies with a number of different molecular genetic approaches have been published phenotyping different PGC-1α-deficient murine strains in the past decade (i.e., complete/incomplete whole-body, organ- or cell type-specific, and conditional/germline knockout strains). These together delineated a phenotype rather reminiscent of a mitochondrial disease, with prominent spongiform intramyelin brain vacuolation, brainstem and cerebellar gliosis, and Purkinje cell loss in the CNS, accompanied by mild myopathic changes in the skeletal muscle (Lucas et al., 2012, 2014b; Szalardy et al., 2013, 2016a,b). These neuropathological hallmarks are accompanied by reduced locomotion, muscle weakness, and ataxia (Lucas et al., 2012, 2014b; Szalardy et al., 2016a), a motor phenotype intriguingly not found to progress with age (Lucas et al., 2012; Szalardy et al., 2016a). Despite the fact that most of the above listed human CNS disorders associate with non-motor features, only few studies have so far addressed non-motor phenotypic alterations in PGC-1α-deficient animals, providing in part contradictory results from different knockout strains, with each focusing on particular aspects (Leone et al., 2005; Agudelo et al., 2014; Lucas et al., 2014a; Bartley et al., 2015). On the basis of this, our current study aimed at revisiting anxiety-related behavior (by means of three independent methods), depression-related behavior, and visuospatial memory in relatively large cohorts of whole-body full-length (FL-)PGC-1α-deficient mice, with special focus on the potential influence of age and sex. Based on the recent findings of altered expression of KATs (the enzymes responsible for KYNA synthesis) in PGC-1α-deficient and transgenic PGC-1α-overexpressing tissues and its proposed role in the anhedonic phenotype of muscle-specific PGC-1α deficient mice (Agudelo et al., 2014), an additional focus has been placed on the qualitative analysis of KYNA production in different tissues and brain regions of FL-PGC-1α-deficient mice. Our results reveal an intriguing fearless but depressive-like phenotype of non-progressive nature, with no overt signs of hippocampal memory involvement and no evidence for an altered KYNA synthesis. The potential clinicopathological correlations are discussed.

## Materials and Methods

### Behavioral Analysis

#### Animals

For behavioral studies, male and female FL-PGC-1α -/- (*n* = 26–37 per cohort) and C57Bl/6J wild-type (*n* = 34–42 per cohort) mice were used. These knockout mice (Leone et al., 2005) lack the expression of the FL-PGC-1α protein but express an N-terminal fragment of 254 amino acids (Chang et al., 2012), and have recently been comprehensively characterized in terms of neuropathological alterations and motor phenotype (Szalardy et al., 2013, 2016a,b). Similarly to that reported before (Szalardy et al., 2016a), the experimental animals of each study cohort were randomized from a population ranging through a wide spectrum of age (12–91 w, overall medians: 48.6 w FL-PGC-1α -/- vs. 47.9 w wild-type) to create age- and sex-matched wide-age-range cohorts. The FL-PGC-1α -/- mice strain developed on C57Bl/6J background were originally generated in the Kelly Lab (Sanford-Burnham Institute for Medical Research at Lake Nona, Orlando, FL, United States) and the founders of the FL-PGC-1α -/- and +/+ populations bred in our institute were a kind gift from Albert C. Ludolph and Patrick Weydt from the Department of Neurology, University of Ulm, Ulm, Germany, as littermates born from +/- breeding pairs. The genotyping of these animals was carried out as previously described by Leone et al. (2005). Keeping in mind the previously reported unexpected loss of PGC-1α -/- pups (Lin et al., 2004), in order to achieve sufficient subject numbers for the large-scale analyses, the experiments were conducted on first-, second-, and third-generation offsprings of FL-PGC-1α -/- and +/+ populations resulted from homozygous × homozygous mating of the founders, bred under identical conditions, within the same room, handled by the same staff. The animals were housed in cages (maximum 4 per cage) in standard conditions with 12–12-h light-dark cycle and free access to water and standard pellet food. Three wide age-range-cohorts were created to assess anxiety-related behavior, depressive-like behavior, and visuospatial memory, respectively (the general characteristics of the experimental cohorts are presented in **Table 1**). The animals were placed in a transport room localized in the immediate vicinity of the experimental room 12 h before the behavioral assessments in order to adapt to the experimental conditions. The animals were examined during the same time of the day to minimize the bias of diurnal rhythm. The experimental setups were thoroughly wiped using a solution containing 30% ethanol, and were allowed to dry completely following every single experimental session to prevent bias raising from olfactory cues. Standard light and temperature conditions were provided during the study. The experiments complied with the EU Directive 2010/63/EU for animal experiments and were approved by the local Animal Care Committee.

**Table 1:** Description of experimental cohorts used to asses different behavioral domains.

		FL-PGC-1α -/-	Wild-type	*p*
**Anxiety**				
*n*=		26	34	–
Female/male		14/12	17/17	0.768
Age	w	39.8 [17.9–51.1]	39.7 [21.9–62.5]	0.627
Younger/older		13/13	17/17	
**Despair**				
*n*=		37	42	–
Female/male		18/19	20/22	0.927
Age	w	54.9 [27.6–71.9]	56.0 [45.6–71.6]	0.420
Younger/older		17/20	19/23	
**Spatial memory**				
*n*=		30	30	–
Female/male		15/15	15/15	1.000
Age	w	45.9 [20.1–51.9]	42.0 [41.9–64.1]	0.477
Younger/older		14/16	15/15	


*w, weeks*.

#### Anxiety-Related Behavior

To assess potential anxiety-related behavioral alterations, the experimental animals were examined by three different experimental modalities: the OF test, the EPM test, and the LDB test.

The OF test was performed to assess the propensity of the animals to avoid the central zone of the setup (a.k.a. thigmotaxis), an anxiety-related feature previously described to be characteristic of this strain (Leone et al., 2005). The setup comprised a black box with a wall height of 36 cm and an open ambulatory field of 48^∗^48 cm. The experiments were performed under indirect dim light (2.5 lux). The study animal was placed into the center of the field and allowed to spontaneously explore for a duration of 30 min. The pattern of locomotion was recorded by infrared led beams and automatically analyzed by the Conducta 1.0 software linked to the setup (Conducta 1.0 System, Experimetria Ltd., Hungary). In a symmetrical 9-square division (3 × 3 square grid), the relative time spent in the 8 peripheral zones altogether (peripheral/total time %) were used for the statistical analysis as a measure of thigmotaxis. Ancillary parameters such as rearing time (s) and total distance ambulated (cm) were used to validate the findings by comparing to prior results with this strain.

The EPM test was performed on the same cohort 4 weeks after the OF test. The maze comprised four arms of 35 cm in length and 10 cm in a width, with two opposing arms enclosed from the sides by black walls of 20 cm in height (closed arms), whereas the other two arms remained unclosed (open arms). The floor of the maze was covered with white plastic film and was located 50 cm above the floor of the room. The whole setup was encompassed by a white belt of 90 cm in height and 120 cm in diameter to create a homogeneous bright arena with no visual bias. The examination was performed under standard diffuse bright light intensity conditions (800 lux). The mice were put in the central area (with head to an open arm distal from the examiner) and let freely explore for 5 min. The locomotion pattern of the animal was tracked and recorded by a video-associated behavioral tracking system (SMART v2.5, Panlab, Spain). The percentage of the time spent in the closed arms (%) was used for statistical analysis. Ancillary parameters included the total distance ambulated (cm) and the distances ambulated in the open or closed arms relative to the time spent within them (cm/s; open velocity, closed velocity).

The LDB test was performed 10 days after the EPM. The apparatus consisted of a 20^∗^30-cm chamber open from above and a 20^∗^20-cm fully covered chamber, interconnected with a 6^∗^6-cm orifice. The experiment was conducted under bright light intensity conditions (1650 lux in the light chamber, <3 lux in the dark chamber). The animal was placed in the light chamber and let freely explore for 3 min. The motion of the experimental animal was tracked by the Smart v2.5 system (Panlab), and the relative time spent in the dark chamber (%) was used for statistical analysis. Ancillary parameters included the distance ambulated in the light chamber relative to the time spent within the light chamber (cm/s; light velocity).

#### Depressive-Like Behavior

The TST, a widely used behavioral paradigm of despair, was used to assess a potential depression-related behavioral alteration due to whole-body FL-PGC-1α deficiency in another study cohort of animals. The examined animal was hanged by the tail with a special clip developed to achieve a firm but soft and pain-free fixation, and were placed into a 40^∗^30^∗^20-cm box. We used a cone-shaped shield to prevent climbing behavior. The tracking duration of 3 min was shorter than the commonly applied 5–6 min to minimize the bias due to decreased endurance. The box was lit from behind through an opaque translucent wall, resulting in a diffuse light inside the closed box. The animals were observed and rated by the experimenter using a video-observer system that recorded the animals through a small hole in the wall of the box, with its size corresponding to that of the lens of the camera. Total time spent in immobility (s) was measured by the experimenter using a stopwatch, and was used for statistical analysis. The animals in this cohort were phenotyped for grip strength 3 months prior to the TST with a method previously described (Szalardy et al., 2016a), and based on the non-progressive feature of weakness in these mice, its measure was used to control for the potential confounding effect of muscle weakness in the observed immobility.

#### Visuospatial Memory

To assess the visuospatial memory performance of FL-PGC-1α -/- mice, the standard two-trial spatial Y-maze test was used, with slight modifications. The method takes advantage of the innate propensity of mice to prefer the novel environment during spontaneous exploration. The maze consisted of a long arm (with a length of 45 cm) that continued in two short arms (with lengths of 36–36 cm) that were perpendicular to each other and made 135–135° angles with the long arm, symmetrically. All three arms were 12 cm in width and were enclosed by transparent plastic walls of 30 cm in height. The maze was located on a board fully encompassed by black walls of 70 cm in height to create an isolated visual environment for the maze. The three arms of the maze were surrounded by unique 2D and 3D visual cues using black and white patterns. A standard dim light was applied during the experiment (70 lux). The activity of the animal was tracked by the Smart v2.5 system (Panlab). The two-trial test comprised an acquisition trial and a recognition trial. During the acquisition trial, the animal was placed into the end of the long arm with head to the wall and were allowed to freely explore the long arm and one of the short arms, with the other one being closed at entry (the animals were randomly assigned to be ‘left-sided’ or ‘right-sided’). The acquisition trial was stopped manually when the study animal spent a total of 2 min within the open short arm. After an inter-trial interval of 30 min, the recognition trial was initiated, where both short arms were open for exploration. The tracking was stopped after the animal reached a total time of 2 min spent in the (known and the novel) short arms together. The ratio of the distance traveled in the novel short arm versus that in both the novel and the known short arms (i.e., recognition distance index) were used for statistical analysis.

### Histological Work-Up

To assess potential between-sex differences in terms of hallmark neuropathological alterations, 4-μm-thick sagittal sections of paraffin-embedded brain halves of 7 female and 8 male FL-PGC-1α-deficient mice sacrificed at 30 w of age were evaluated in different levels. We did not use wild-type specimens for this between-sex analysis as they do not demonstrate pathological vacuolation at this age (Szalardy et al., 2013). We also did not use older animals as myelin vacuolation is part of the ‘physiological’ aging which may serve as a bias for analysis. Various brain regions were evaluated for myelin vacuolation, a core neuropathological feature of PGC-1α-deficient mice, by means of a semi-quantitative method described previously (Szalardy et al., 2013). In brief, Hematoxylin-Eosin and Klüver–Barrera (Luxol and Fast red) stainings were used for the evaluation with the following semi-quantitative scoring scheme used at 40× magnification under the light microscope: (0) No vacuolation: ≤1 vacuole-like alteration per visual field; (1) Scattered vacuolation; 2–3 vacuoles per visual field; (2) Mild vacuolation: 4–10 vacuoles per visual field; (3) Moderate vacuolation: 11–20 vacuoles per visual field; (4) Severe vacuolation: >20 vacuoles per visual field.

### HPLC Measurements

High-performance liquid chromatography (HPLC) was used for the quantitative analyses of TRP and KYNA levels in different murine tissues. The tissue specimens were obtained immediately after decapitation on ice, and kept in sterile polypropylene tubes at -80°C until homogenization. Liver samples of 16 wild-type and 15 FL-PGC-1α-deficient female and male mice and samples of different brain regions of particular involvement in PGC-1α-deficiency (striatum and cerebellum) of 8 wild-type and 6 FL-PGC-1α-deficient male mice were used for the analysis. On the basis of previously reported alterations of TRP levels in the brain in rats (Carlsson and Carlsson, 1988) and mice (Kim et al., 2005) (with female animals having been reported to have slightly though significantly higher levels), only male tissues were used for the analysis of brain specimens. The specimens were weighed and then homogenized for 1.5 min in 250 μl freshly prepared ice-cold solution, containing trifluoroacetic acid (0.1% v/v) and 2 μM 3-nitro-L-tyrosine (3-NLT), as internal standard. The homogenates were centrifuged at 13709 RCF (12000 RPM) for 10 min at 4°C. The resulting supernatants were stored at -80°C until further use. Subsequently, the supernatants were measured with an Agilent 1100 HPLC system (Agilent Technologies, Santa Clara, CA, United States) combined with a fluorescence (FLD) and a UV detector. The FLD was set at excitation and emission wavelengths of 254 and 398 nm; and 344 and 398 nm, respectively, for the quantification of TRP and KYNA. The UV detector was set at 365 nm for the determination of 3-NLT. Chromatographic separations were performed on a Kinetex C18 column, 150^∗^4.6 mm I.D., 5-μm particle size (Phenomenex Inc., Torrance, CA, United States) preceded by a Security Guard pre-column C18, 4^∗^3.0 mm I.D. (Phenomenex Inc.). The mobile phase, containing 0.2 M zinc acetate/ACN = 95/5 v/v%, in which pH was adjusted to 6.2 with acetic acid, was delivered at a flow rate of 1.2 ml/min. The injection volume was 50 μL. As for the validation of the method, the following parameters are reported, briefly, regarding TRP and KYNA. The limit of detection (LOD) and lower limit of quantification (LLOQ) were 10 and 0.4 nM; and 20 and 1 nM, respectively. With regard to precision, the relative standard deviations for the peak area response was ≤2.2%, whereas for the retention time it was ≤0.1%, for both the investigated compounds. The recoveries for the brain samples ranged from 86 to 91% and 82 to 92% for TRP and KYNA, respectively.

### Statistical Analysis

**FIGURE 1:**
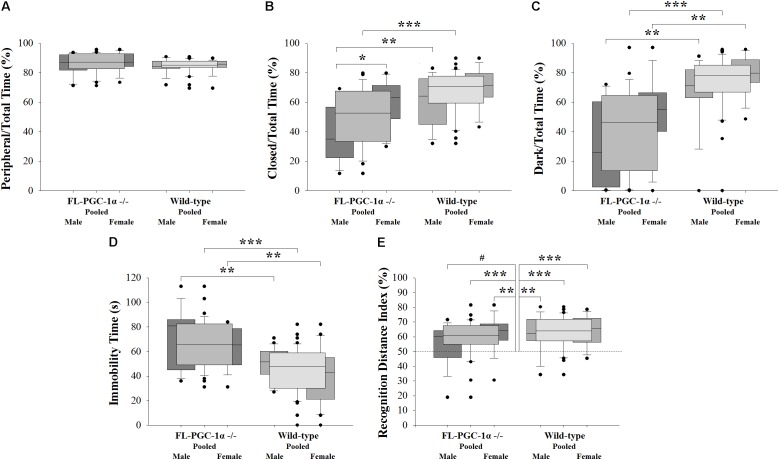
Assessment of psychomotor alterations of FL-PGC-1α-deficient mice. The OF did not reveal signs of thigmotaxis **(A)**, with the EPM **(B)** and LDB **(C)** consistently demonstrating decreased anxiety, with a tendency to be driven predominantly by males. The assessment of despair in the TST revealed significantly increased immobility time irrespective of sex **(D)**, with no evident correlation with muscle strength (not shown). The 2-trial asymmetric spatial recognition Y-maze revealed no overall impairment in spatial memory in FL-PGC-1α-deficient mice, with only a tendency for males to be slightly affected **(E)**. ^∗^*p* < 0.05; ^∗∗^*p* < 0.01; ^∗∗∗^*p* < 0.001; ^#^*p* < 0.065 (regarded as a non-significant trend).

The statistical analysis was performed by SPSS 22.0 software (SPSS Inc., Chicago, IL, United States). The distribution of the variables within each study dataset was assessed by the Shapiro–Wilk test. Variables with normal distribution were analyzed by parametric, whereas those with non-normal distribution by non-parametric tests. Parametric comparative analysis between two groups was performed by Student’s unpaired *t*-test using also the Levene’s test to assess the equality of variances and the Welch’s correction where appropriate. Non-parametric comparative analysis between two groups were performed by the Mann–Whitney *U* test. Correlation analyses were subjected to Pearson’s correlation or Spearman’s rank correlation for variables with normal and non-normal distribution, respectively, with *p*-values corrected for the number of variables within the analysis to avoid family wise error. When controlling the analyses for potential confounders (e.g., age, sex, grip strength performance, and/or body weight), the estimation of the effect sizes of variables were performed by binary logistic regression analyses. On assessing phenotypic progression, subgroups of the younger and older animals were analyzed (split at the median, with subjects having an age corresponding to the median regarded consistently as old; **Table 1**). Multiple comparisons of pre-defined subgroups based on age or sex were performed by one-way analysis of variance (ANOVA) or one-way Kruskal–Wallis test, depending on the distribution of the data, followed by pairwise corresponding *post hoc* analyses with *p*-values corrected for multiple comparisons with the Bonferroni method. The significance of novel place recognition in the Y-maze was tested by the one-sample *t*-test or the one-sample Wilcoxon signed rank test in the case of normal or non-normal distribution, respectively, setting the reference level to 50% (i.e., zero preference). The analysis of discrete variables obtained from the semi-quantitative scoring of histological preparations were performed by the Fisher’s exact test. Data are presented as mean (± standard error of mean) or median [interquartile range] for variables with normal and non-normal distribution, respectively. A *p*-value less than 0.05 was regarded as significant. Data of behavioral analyses in **Figure 1** are presented in box plots separately for sexes that are superimposed by the box plot of the corresponding pooled data, enabling the visualization of sex- and genotype-related effects, respectively.

## Results

### Assessment of Anxiety-Related Behavior

Three consecutive experiments with different behavioral modalities were performed to assess potential anxiety-related alterations in FL-PGC-1α -/- mice. In the pooled analysis, the OF test revealed no significant differences between FL-PGC-1α -/- and wild-type mice in terms of their propensity to avoid the central zone (*p* = 0.092; 87.3% [82.9–92.9%] vs. 85.3 [83.6–87.9%]; **Figure 1A**), indicating no signs of thigmotaxis. Likewise, the peripheral/total time % was not a significant predictor of the genotype in the binary logistic regression analysis controlling for age and sex [*p* = 0.133, Wald = 2.258, Exp(*B*) = 0.922 (95% confidence interval (CI) = 0.830–1.025]. No significant differences were found either between sexes within genotypes or between genotypes within sexes (*p* > 0.05 for all *post hoc* analysis). This measure did not correlate with age and no differences were found between young and elderly subgroups in either genotype (*p* > 0.05 for all). In the pooled analysis, the OF paradigm also revealed significantly decreased ambulation distance and rearing time in FL-PGC-1α -/- mice (*p* < 0.001 for both ancillary variables), with effect sizes in the logistic regression analyses controlled for age and sex being comparable to that reported in our prior publication [ambulation distance: *p* < 0.001, Wald = 13.078, Exp(*B*) = 1.0007 (95% CI = 1.0003–1.0010); rearing time: *p* < 0.001, Wald = 11.529, Exp(*B*) = 1.028 (95% CI = 1.012–1.045)], recapitulating previous findings (Szalardy et al., 2016a).

Having demonstrated no signs indicative of thigmotaxis, the cohort was subjected to paradigms being more specific for anxiety. Intriguingly, the pooled analysis of the EPM paradigm not only failed to detect anxiety in FL-PGC-1α -/- compared to wild-type mice, but demonstrated significantly less anxiety-related behavior in this genotype compared to wild-type (closed/total time %: *p* < 0.001, 52.7% [33.4–67.6%] vs. 70.5% [59.5–77.8%]; **Figure 1B**). The effect of this variable in predicting the genotype was highly significant when controlled for age and sex [*p* < 0.001, Wald = 10.689, Exp(*B*) = 1.068 (95% CI = 1.027–1.111], corresponding to a 6.8% increase in the odds of being wild-type with every additional % elevation in the relative time spent in the closed arms. This difference between genotypes tended to be more pronounced among male mice (*p* = 0.008), having lost significance among females following Bonferroni correction (*p* = 0.176). A similar dichotomy was reflected also by the different effect sizes obtained from sex-wise logistic regression analyses controlled for age [*p* = 0.032, Wald = 7.052, Exp(*B*) = 1.079 (95% CI = 1.020–1.142) for males; *p* = 0.264, Wald = 3.379, Exp(*B*) = 1.058 (95% CI = 0.096–1.124) for females]. Within FL-PGC-1α -/- mice, this low-anxiety feature was significantly more pronounced in male mice compared to females (*p* = 0.017), with no such between-sex difference observed among wild-types (*p* = 0.424). However, due to a substantial overlap between the 95% CI of the Hodges–Lehman estimators of the median of difference, the difference between the observed differences in a given comparison (irrespectively of whether they are in fact significant or not) is not statistically significant at α = 5%; therefore, we cannot draw firm conclusions on either the male-specificity of the observed between-genotype difference or the genotype-specificity of the between-sex difference. As regards phenotypic progression, no significant correlation was observed with age in any groups, along with no significant differences between young and elderly subgroups (*p* > 0.05 for all), indicating a non-progressive nature of the observed alteration in FL-PGC-1α -/- mice. Differences regarding ancillary measures such as total and time-relative distances ambulated (i.e., ‘velocities’) were not statistically significant between genotypes (not shown).

This pattern was almost completely recapitulated by a subsequent experimental paradigm in the LDB performed with the same cohort, revealing significantly less relative time spent in the dark chamber in FL-PGC-1α -/- mice compared to wild-type animals (dark/total time %: *p* < 0.001, 46.1% [13.5–64.6%] vs. 78.2% [66.8–85.2%]; **Figure 1C**). This variable was also a strong predictor of genotype when controlled for age and sex, with a similar effect size to that of the closed/total time % in the EPM [*p* < 0.001, Wald = 15.532, Exp(*B*) = 1.076 (95% CI = 1.038–1.116)], corresponding to a 7.6% increase in the odds of being wild-type with every additional % elevation in the relative time spent in the dark. The difference between genotypes was consistent and significant in both sexes even after Bonferroni correction (*p* = 0.004 for both sexes), with comparable effect sizes when controlled for age [*p* = 0.020, Wald = 7.839, Exp(*B*) = 1.064 (95% CI = 1.019–1.111) for males; *p* = 0.025, Wald = 7.491, Exp(*B*) = 1.096 (95% CI = 0.126–1.170) for females]. In this case, no significant between-sex differences were observed in either the FL-PGC-1α -/- (*p* = 0.192) or the wild-type subcohorts (*p* = 0.292). No significant correlation was observed with age in any groups, along with no significant differences between young and elderly subgroups (*p* > 0.05 for all), likewise indicating a non-progressive alteration in FL-PGC-1α -/- mice. The analysis of ancillary measures revealed significantly decreased ambulation distance relative to the time spent in the light chamber (light velocity) in FL-PGC-1α -/- mice compared to wild-types [*p* < 0.001, 3.2 (±0.3) cm/s vs. 5.0 (±0.3) cm/s] irrespective of sex.

To estimate the concordance of the observed data within the total experimental cohort, correlation analyses were performed by the use of the core variables of the three anxiety-related measures. These revealed that the performance of the animals in the EPM (closed/total time %) significantly correlated with that in the LDB (dark/total time %; *p* = 0.012, Spearman’s Rho = 0.386), whereas the peripheral/total time % the OF (as a measure of thigmotaxis) did not correlate with either variables (*p* = 1.000, Spearman’s Rho = 0.035 with closed/total time %; *p* = 1.000, Spearman’s Rho = 0.021 with dark/total time %). Furthermore, ancillary parameters of general ambulatory behavior (absolute distances traveled during tracking) measured along the different modalities were significantly and remarkably strongly concordant among FL-PGC-1α -/- animals (but not among wild-types), with the majority demonstrating significant correlations with the strongest low-anxiety parameter (dark/total time %) as well (**Supplementary Table S1**).

Controlling for the potential though unlikely bias of general hypomotility underlying a specific regional preference, we found that the effect sizes of the EPM and LDB outcomes did not significantly change when potential confounder variables from either independent (ambulation distance from the OF) or dependent modalities (total distances ambulated in the EPM or in the light chamber of LDB) were controlled for in a variety of binary logistic regression models alone or in combination.

### Assessment of Depressive-Like Behavior

The pooled analysis of the TST paradigm revealed significantly more time spent in immobility in FL-PGC-1α -/- mice compared to wild-type animals [*p* < 0.001, 67.5 (±3.2) s vs. 45.0 (±2.8) s, **Figure 1D**]. The variable was a strong predictor of genotype when controlled for age and sex, [*p* < 0.001, Wald = 16.378, Exp(*B*) = 0.936 (95% CI = 0.907–0.967)], corresponding to a 6.4% decrease in the odds of being wild-type with every additional s elevation in the time spent in immobility. The difference between genotypes was consistent and significant in both sexes even after Bonferroni correction (*p* = 0.007 for males, *p* = 0.001 for females), with comparable effect sizes as a significant predictor of the genotype when controlled for age [*p* < 0.005 for both; Exp(*B*) = 0.939 and 0.930 for males and females, respectively]. In this case, no significant between-sex differences were observed in either the FL-PGC-1α -/- (*p* = 1.000) or the wild-type subcohorts (*p* = 0.304). No significant correlation was observed with age in any groups, along with no significant differences between young and elderly subgroups (*p* > 0.05 for all), indicating a non-progressive alteration in FL-PGC-1α -/- mice. Within the FL-PGC-1α -/- genotype, the performance in the TST did not show a significant correlation with their prior performance in the inverted screen (*p* = 1.000, Spearman’s Rho = -0.045), suggesting that the observed immobility behavior is not merely attributable to decreased endurance, a feature characteristic of these animals. Likewise, the entry of this variable did not significantly influence the effect size of immobility time in predicting the genotype, similarly to weight, which was not significantly different between groups in the present analysis (not shown).

### Assessment of Visuospatial Memory Performance

The pooled analysis of the spatial Y-maze paradigm revealed no significant difference in the recognition performance of FL-PGC-1α -/- mice compared to wild-type animals (recognition distance index %: *p* = 0.274, 60.9% [55.0–67.6%] vs. 64.0% [57.3–72.0%]; **Figure 1E**). The variable did not predict the genotype when controlled for age and sex either [*p* = 0.158, Wald = 1.990, Exp(*B*) = 31.005 (95% CI = 0.263–3661.051)]. Both genotypes significantly preferred the novel arm in the one-sample analyses when pooled for sexes (*p* < 0.001 for both genotypes). In the sex-wise analysis; however, male FL-PGC-1α -/- mice failed to demonstrate significant preference (*p* = 0.061, median of difference = 7.3%; 95% CI = -0.8–13.8%), while female FL-PGC-1α -/- mice (*p* = 0.006) along with both the male (*p* = 0.005) and the female (*p* < 0.001) wild-type subgroups demonstrated firm recognition. Nevertheless, there were no statistically significant differences between genotypes within sexes (including between male FL-PGC-1α -/- and male wild-type mice) or between sexes within genotypes (*p* > 0.05 for all). On the basis of the lack of between-subgroup differences and that the observed data in male FL-PGC-1α -/- mice is statistically consistent with a possible true median recognition distance index % up to 63.8% (based on the 95% CI), we cannot draw a firm conclusion on whether male FL-PGC-1α -/- mice indeed have an impairment in visuospatial memory. No significant correlation was observed with age in any groups, along with no significant differences between young and elderly subgroups (*p* > 0.05 for all).

### Between-Sex Histopathological Analysis

Observing tendencies in the EPM and in the Y-maze paradigms for FL-PGC-1α -/- males to be slightly more affected, supplementary histopathological semi-quantitative comparative analysis was performed between sexes as regards the extent of myelin vacuolation, a core neuropathological alteration in PGC-1α -/- mice, in various brain regions. The results revealed no statistically significant differences in any examined brain regions, revealing at most non-significant tendencies for a male preponderance especially in the pontomedullary brainstem and the motor cortex in the region-wise analysis; however, a nominally significant though rather slight overall difference could be detected between the mean vacuolation scores of all analyzed brain regions in the pooled analysis, suggesting a minimal overall predominance in males (2.3 ± 0.1 vs. 2.1 ± 0.1, *p* = 0.036). The detailed findings are summarized in **Table 2**.

**Table 2:** Assessment of between-sex differences in the semi-quantitative scores of brain vacuolation in FL-PGC-1α-deficient mice in selected brain regions.

Vacuolation	Male	Female	*p*
Anterior Commissure	2.9 ± 0.3	2.1 ± 0.1	0.152
Hippocampus^med^	1.0 ± 0.3	1.3 ± 0.3	1.000
Hippocampus^lat^	1.9 ± 0.5	2.0 ± 0.0	1.000
Thalamus^med^	2.5 ± 0.3	1.6 ± 0.2	0.236
Thalamus^lat^	3.5 ± 0.3	3.6 ± 0.2	1.000
Nucleus accumbens	3.0 ± 0.4	2.1 ± 0.3	0.190
Mammillary body	1.7 ± 0.5	2.6 ± 0.3	0.592
Mesencephalon^med^	2.4 ± 0.2	2.6 ± 0.3	1.000
Mesencephalon^lat^	3.0 ± 0.0	2.5 ± 0.5	0.333
Pontomedullary brainstem^med^	3.3 ± 0.2	2.6 ± 0.2	0.058
Pontomedullary brainstem^lat^	2.1 ± 0.1	1.7 ± 0.5	0.054
Cerebellar cortex^med^	0.3 ± 0.2	1.1 ± 0.2	0.125
Cerebellar cortex^lat^	0.3 ± 0.2	0.3 ± 0.2	1.000
Cerebellar nuclei^med^	2.3 ± 0.3	2.0 ± 0.0	1.000
Cerebellar nuclei^lat^	2.3 ± 0.3	2.0 ± 0.4	1.000
Cerebellar white matter^med^	1.8 ± 0.3	2.0 ± 0.2	0.765
Cerebellar white matter^lat^	2.0 ± 0.2	1.6 ± 0.4	0.648
Fasciculus retroflexus	4.0 ± 0.0	4.0 ± 0.0	1.000
Motor cortex	2.0 ± 0.0	1.4 ± 0.5	0.054
Sensory cortex	2.9 ± 0.1	2.3 ± 0.2	0.103
Visual cortex	2.6 ± 0.2	2.6 ± 0.2	1.000
Insular cortex	2.2 ± 0.2	1.8 ± 0.2	1.000
Striatum	4.0 ± 0.0	4.0 ± 0.0	1.000
Globus pallidus	2.7 ± 0.3	2.7 ± 0.3	1.000
Amygdala	1.4 ± 0.7	0.8 ± 0.3	0.420
Stria terminalis	2.7 ± 0.3	2.9 ± 0.3	1.000
Internal capsule	3.5 ± 0.2	3.7 ± 0.2	0.592
Fimbria hippocampi	1.8 ± 0.3	0.9 ± 0.3	0.273
Optic tract	0.3 ± 0.2	0.5 ± 0.3	1.000
Olfactory tract	2.0 ± 0.4	2.1 ± 0.3	0.767


### Measurement of KYNA and TRP Levels

On the basis of a prior report on altered KAT expression in muscle-specific PGC-1α-deficient and transgenic PGC-1α overexpressing mice (Agudelo et al., 2014), HPLC analysis of KYNA and TRP was performed in liver, cerebellar, and striatal specimens. The results demonstrate no significant differences between groups in either metabolites or the KYNA/TRP ratio in any examined tissues, and are summarized in **Table 3**.

**Table 3:** Assessment of the effect of FL-PGC-1α-deficiency on kynurenic acid synthesis in the murine liver and brain (HPLC).

		FL-PGC-1α -/-	Wild-type	*p*
**Liver**				
*n*=		15	16	–
Female/male		7/8	8/8	0.853
KYNA	pmol/g ww	154.4 [0.0–173.6]	144.6 [122.2–271.8]	0.495
TRP	nmol/g ww	193.2 [61.6–418.2]	170.6 [117.9–398.6]	0.470
KYNA/TRP	%	0.09 [0.00–0.23]	0.09 [0.02–0.22]	0.892
**Brain**				
*n*=		6	8	–
Female/male		0/6	0/8	1.000
KYNA striatum	pmol/g ww	41.9 [0.0–157.5]	0.0 [0.0–143.8]	0.662
TRP striatum	nmol/g ww	31.8 ± 6.2	22.7 ± 1.5	0.205
KYNA/TRP striatum	%	0.10 [0.00–0.77]	0.00 [0.00–0.61]	0.852
KYNA cerebellum	pmol/g ww	18.0 ± 9.1	27.7 ± 8.2	0.448
TRP cerebellum	nmol/g ww	42.2 ± 6.2	41.2 ± 6.1	0.908
KYNA/TRP cerebellum	%	0.02 [0.00–0.12]	0.06 [0.02–0.22]	0.573


*KYNA, kynurenic acid; TRP, tryptophan; ww, wet weight.*

## Discussion

A number of reports have been published describing certain aspects of locomotor alterations in PGC-1α-deficient mice, and recently, comprehensive works have together circumscribed a motor phenotype of decreased locomotion, muscle weakness, and ataxia, in association with the absence of either PGC-1α or FL-PGC-1α in systemic and CNS-specific knockouts (Lucas et al., 2012, 2014b; Szalardy et al., 2016a). Despite the fact that PGC-1α-deficiency has been linked to various neurodegenerative disorders that are associated with psychic alterations (Rona-Voros and Weydt, 2010; Szalardy et al., 2015) and also with schizophrenia (Jiang et al., 2013; McMeekin et al., 2016) and the fact that a GWAS study has linked the chromosomal localization of the PGC-1α gene (*PPARGC1A*, 4p15.1-2) to schizophrenia and bipolar disorder (Christoforou et al., 2007), only a few studies have addressed psychic behavioral alterations in the literature. In addition, these dealt only with specific aspects, such as anxiety-related behavior (Leone et al., 2005), depression-related behavior (Agudelo et al., 2014), and certain hippocampal functions (Lucas et al., 2014a; Bartley et al., 2015), and the analyses were performed in different PGC-1α knockout strains, evaluating either only males or females and males in a pooled manner (**Table 4**). As none of these findings have so far been recapitulated in the literature and some of them have raised intriguing questions, our aim was to revisit the observations of anxiety-like behavior, despair, and hippocampal impairment in whole-body FL-PGC-1α-deficient mice bred in our laboratory. On the basis of prior findings that PGC-1α-deficiency-related motor alterations appear to be non-progressive (Lucas et al., 2012; Szalardy et al., 2016a) and the observations that the effect of PGC-1α dysfunction tends to show a male preponderance (Eschbach et al., 2013; Weydt et al., 2014), we performed our analysis on large-subject-number cohorts covering a wide age range, enrolling animals from both sexes in an age-matched manner. An attempt is made to interpret the present data in light of the literature reported on different PGC-1α knockout strains with the inherent limitations considered and discussions made accordingly.

**Table 4:** Previous reports on non-motor behavioral alterations of PGC-1α-deficient and transgenic animals.

Publication	Strain	Behavior	Paradigm(s)	Findings	Age	Sex	Subject number
Leone et al., 2005	Whole-body FL-PGC-1α **-**/**-**	Anxiety	Open-field	Thigmotaxis	3.5 m	Male	*n* = 11 vs. 8
Agudelo et al., 2014	Muscle-specific PGC-1α transgenic	Despair	Forced-swim test	Decreased immobility after CMS, no baseline difference	12–13 w	Male	*n* = 8–10
			Sucrose consumption	Increased after CMS, no baseline difference			
	Muscle-specific PGC-1α **-**/**-**		Sucrose consumption	Decreased (anhedonia)	N/A	N/A	*n* = 7–8
Lucas et al., 2014a	PV+ neuron-specific PGC-1α **-**/**-**	Hippocampal memory	Barnes maze test	Increased distance traveled to reach the escape box	3 (or 6) m	Male	*n* = 11 vs. 6
Bartley et al., 2015	Whole-body PGC-1α **-**/**-**	Hippocampal function	Nest building	Impaired	2 m	Mixed	*n* = 13 vs. 10
	PV+ neuron-specific PGC-1α **-**/**-**			Impaired	2–8 m	N/A	*n* = 6 vs. 8


*CMS, chronic mild stress; m, months; N/A, explicit data not available, w, weeks.*

Among the pioneering publications with PGC-1α-deficient mice, Leone et al. (2005) reported the observation that their whole-body PGC-1α knockout strain (today known as FL-PGC-1α-deficient mice) demonstrated increased avoidance of the central zone in the OF (a.k.a. thigmotaxis), a behavioral alteration interpreted as increased anxiety. Analyzing the results of the 30-min OF test in this current study, our first observation was that FL-PGC-1α-deficiency was not associated with thigmotaxis in any sex or age group. Core locomotor parameters were, however, comparable to that reported previously (Szalardy et al., 2016a). To further address this issue and to verify our results, after abundant periods of recovery, the same cohorts of animals were subjected to more specific tests of anxiety-related behavior, the EPM and LDB. Interestingly enough, the EPM did not only fail to reveal increased anxiety in FL-PGC-1α-deficient mice, but demonstrated a significant decrease in anxiety-related behavior in these animals, especially in males. This pattern was almost completely recapitulated in the LDB.

Though it is unlikely that hypomotility *per se* could concordantly skew the ambulation toward the preference of a given type of area throughout different modalities, we addressed the potential bias originating from hypomotility characteristic of FL-PGC-1α-deficiency in a variety of logistic regression models, revealing no significant influence on the overall effect sizes. The lack of significant influence is also reflected by the fact that hypomotility was not a statistically significant feature of FL-PGC-1α-deficiency in the EPM paradigm (in terms of total distance ambulated) contrasting with the OF and LDB paradigms, still providing concordant results with the LDB in terms of the assessed non-motor behavior. This is also supported by the observation that FL-PGC-1α-deficient mice not only spent less time in the closed arms in the EPM but demonstrated significantly less closed/total distance ambulated as well (not shown), which together with the lack of significantly altered total ambulation distance in the whole setup directly indicates a regional preference without evident hypomotility in this particular paradigm.

To approach the not unprecedented phenomenon that PGC-1α-deficiency tended to affect males more, we performed an extensive sex-wise comparative histopathological analysis as regards myelin vacuolation, a core neuropathological alteration associated with PGC-1α-deficiency in the brain. The overall comparison of subject-wise mean semi-quantitative scores of every single brain region analyzed and scored revealed a nominally significant though rather slight male predominance of this alteration. The mean difference was most pronounced in the pontomedullary brainstem; however, the scores did not show statistical significance in the region-wise analysis due to relatively high deviation. In the presence of a whole-body genetic alteration that is associated with multiple systemic metabolic and CNS-related alterations, an exact clinicopathological correlation with the aim to explain a given phenotypic aspect usually cannot be drawn, as most probably a constellation of endocrine, metabolic, as well as structural and functional CNS alterations can be accounted for the given phenotype. However, based on the observation that the most devastatingly affected structures include the fasciculus retroflexus (a.k.a. the habenula-interpeduncular tract), one might speculate that the disruption of the habenula-interpeduncular connection may at least in part be attributable to the observation. Indeed, lesioning of this pathway or the lateral habenula itself has been repeatedly linked to decreased anxiety in rodents (Murphy et al., 1996; Pobbe and Zangrossi, 2008; Gill et al., 2013; Casarrubea et al., 2015). Interestingly, lesions in this pathway have also been associated with decreased maternal behavior (Matthews-Felton et al., 1995), which is in accordance with our unpublished impression about PGC-1α-deficient females, a phenomenon that may also contribute to the remarkable loss of pups born otherwise in normal average number in the litter (Lin et al., 2004).

As a next step of psychomotor phenotyping, we subjected PGC-1α-deficient animals to the TST test, an established animal model of despair. Our findings depict a clear increase in immobility compared to age- and sex-matched controls, irrespective of age. No significant between-sex difference was observed in this case. These results nicely correspond with those of a recent study revealing decreased sucrose consumption as a sign of anhedonia in muscle-specific PGC-1α-deficient mice and increased sucrose consumption and decreased immobility in the forced swim test after chronic mild stress, another model of despair, in muscle-specific transgenic PGC-1α overexpressing mice (Agudelo et al., 2014). The authors of this comprehensive study also reported significantly decreased expression of KATs in the PGC-1α-deficient animals, enzymes responsible for the synthesis of KYNA from the TRP metabolite, L-kynurenine, and argued that a peripheral downregulation of KYNA synthesis might be responsible for the observed depressive-like behavior in muscle-specific PGC-1α knockouts. Of note, KYNA is a neuroprotective end-metabolite of the kynurenine pathway that poorly penetrates the BBB (Fukui et al., 1991) and whose absolute (Myint et al., 2007; Bay-Richter et al., 2015) or relative levels (compared to that of a toxic kynurenine metabolite, QUIN, in form of a ratio) (Savitz et al., 2015) had indeed been found decreased in the blood of depressed patients. The hypothesis of the authors was based on the rationale that a decreased peripheral synthesis of KYNA would allow the relative overrepresentation of other kynurenine metabolites in the brain that are associated with higher BBB-penetrance and/or presumed pathogenic roles in major depression as neurotoxic and neuroinflammatory factors (Brundin et al., 2016), thereby contributing to the development of depressive-like symptoms in animals with muscle-specific PGC-1α deficiency (Agudelo et al., 2014). No direct measures were, however, published in support of this hypothesis at the metabolite level. Encouraged by our decades of interest in kynurenine pathway-related alterations, especially in relation with neurodegenerative disorders (Zadori et al., 2011; Vecsei et al., 2013; Veres et al., 2015) and neurocognitive alterations (Szalardy et al., 2012; Zadori et al., 2018), we measured TRP and KYNA levels in the liver and different brain regions of whole-body FL-PGC-1α knockout mice. Our results overall do not support that PGC-1α deficiency would significantly alter KYNA synthesis in mice at the metabolite level either in the periphery or in the CNS, and suggest that other causes should be attributable to the observed depressive-like phenotype.

To rule out the potential significant bias of muscle weakness characteristic of these animal strains, we controlled for the performance of these animals in the wire-hang test conducted several weeks earlier (a parameter previously demonstrated not to alter with aging). Of note, the measured time of immobility in the TST and the latency to fall from the grid did not show inverse correlation within the PGC-1α-deficient group, likewise, the ability of the TST performance to distinguish between the genotypes were not altered significantly by the inclusion of the wire-hang performance in a binary logistic regression model, suggesting that muscle weakness on its own cannot be purely responsible for the despair-like phenotype. Despite all this, the contribution of muscle weakness to the observed phenomenon to a certain extent is likely, with the precise value being difficult to judge.

The previous observation that muscle-specific disruption of PGC-1α was likewise associated with anhedonic phenotype irrespective of the muscle function suggests that alterations in circulatory factors should contribute to the observed despair behavior. On this basis, it is tempting to speculate that the observed anhedonic behavior can be also attributed to a form of ‘sickness behavior’ [as presumed also previously (Agudelo et al., 2014)], a phenotype suggested to be mediated by increased circulating levels of IL-6, IL-12, and TNFα penetrating to the brain (Muller and Schwarz, 2007). Of note, PGC-1α has been shown to potently decrease the expression of IL-12 in the murine skeletal muscle (Eisele et al., 2015). Furthermore, a marked elevation of IL-6 and TNFα have been reported in the serum of muscle-specific PGC-1α-deficient mice, together with a strong inverse correlation of PGC-1α mRNA levels with IL-6 and TNFα gene expression in diabetic human muscles (Handschin et al., 2007). In addition, PGC-1α overexpression has recently been shown to downregulate IL-6 expression in astrocytes (Nijland et al., 2014). These together indeed raise the possibility of a ‘sickness behavior’ underlying the observed despair, however, the involvement of multiple brainstem structures relevant in the pathogenesis of depression in the neuropathology present in these mice definitely renders the identification of the most prominent cause difficult.

The fact, however, that an effect associates with anxiolytic-like together with depressive-like patterns is peculiar and unexpected *per se*, as these tests most often present concordant findings. Only few publications can be found reporting similar discordance. In particular, forebrain-specific disruption of GR in mice lead to similar anxiety-like performance in the EPM and LDB tests in association with increased despair in the TST and forced swim tests, with likewise no alterations in the OF whatsoever, based on which the authors concluded that different pathways should mediate these behaviors (Boyle et al., 2006). Interestingly enough, GR and PGC-1α proteins have been reported to directly and physically interact with each other, e.g., forming functional complexes when enhancing the transcription of PEPCK (a gluconeogenesis gene) (Yoon et al., 2001; Herzog et al., 2004) or repressing the transcription of the insulin gene (Jang et al., 2007). In fact, PGC-1α was first discovered in humans as a potent tissue-specific co-activator of the GR (originally referred to as LEM6) (Knutti et al., 2000). In addition, glucocorticoids (such as dexamethasone) are among the most potent inducers of PGC-1α expression (Knutti et al., 2000; Yoon et al., 2001; Herzog et al., 2004), further underlying the relevance of a PGC-1α-GR positive regulatory loop in stress-induced responses, likely including stress-related behavior. Whether and to what extent the disruption of this prominent functional connection inside the CNS could be responsible for the recapitulation of the intriguing phenotype of forebrain GR-deficient mice, especially in light of the several structural alterations in the brain of FL-PGC-1α-deficient mice, is at present unknown.

Finally, a cohort of FL-PGC-1α-deficient mice were subjected to the two-trial spatial recognition Y-maze test, an established and sensitive model for spatial memory in rodents (Dellu et al., 1992, 2000). Previously, PV-positive neuron-specific male PGC-1α knockout animals have been reported to exhibit deficits in the Barnes maze test in form of a longer distance traveled to find the escape box on day 5 after daily training, suggesting deficits in long-term spatial memory formation, a form of hippocampus-dependent learning (Lucas et al., 2014a). Subsequently, the same group reported deficits observed in nest building, an innate hippocampus-related behavior, in both the whole-body as well as PV-positive neuron-specific PGC-1α knockout mice, with no differences reported between males and females, together indicating impairment in hippocampus-dependent functions irrespective of sex (Bartley et al., 2015). These findings were proposed to be not related to hypomotility as, intriguingly, PV-positive conditional neuron-specific PGC-1α-deficient mice reported not to exhibit motor impairment (Lucas et al., 2014a; Bartley et al., 2015). Our results in the two-trial Y-maze test are not concordant with these prior publications, however, with no between-cohort differences observed. In fact, FL-PGC-1α-deficient mice, especially females, provided fairly high median preference of the novel arm in the retrieval trial. A limitation of interpretation is that male FL-PGC-1α-deficient mice showed somewhat poorer preference, secondary to higher variability in this group. It is possible that a higher subject number could have powered up our analysis to provide statistically significant difference in males; however, we were unfortunately not able to set up a single cohort at the same time with a higher subject number, which was already 2–5-times larger than previous studies reporting hippocampus-based behavioral involvement in PGC-1α-deficient mice at the cohort level. The excellent recognition of females and the high upper value of the 95% CI in males allow as to conclude that FL-PGC-1α-deficiency in general is not associated with significant impairment in spatial memory formation. Whether the genotype difference between FL-PGC-1α-deficiency and complete PGC-1α-deficiency, i.e., the residual activity of the NT-PGC-1α itself, was capable of rescuing the phenotype is at present unclear. This in general intact visuospatial recognition also allows as to suggest that the performance of FL-PGC-1α-deficient animals in other paradigms are not biased by possible visual impairment, as supported also by the largely intact optic nerves (**Table 2**) and retinal structure (Szalardy et al., 2016a) in these animals.

## Conclusion

The present study revisited prior observations of anxiety-related, depression-related, and hippocampal memory-related observations made on different PGC-1α-deficient murine strains, in a large-subject-number analysis on whole-body FL-PGC-1α-deficient mice. This study is the first in the literature to address all these non-motor behavioral domains in a single PGC-1α-deficient strain, with special focus on the potential effect of age and sex. The findings revealed no signs of previously reported anxiety-related behavior, but revealed an unexpected, anxiolytic-like phenotype, consistent throughout different paradigms, with a slight male preponderance. This was associated with despair-like anhedonic behavior, consistent with that reported previously, but did not associate with either peripheral or CNS-related alterations in KYNA synthesis, which was previously proposed. Though male FL-PGC-1α-deficient mice tended to perform poorer in a hippocampus-based spatial learning paradigm, the genotype overall was not associated with impairment in spatial memory, contradicting with prior observations. None of the observed alterations showed age-dependence, similarly to motor alterations as reported previously (Szalardy et al., 2016a). In light of the substantial multi-systemic involvement of PGC-1α deficiency in mice, the provision of exact etiological explanation for the observed behavioral alterations could not be attempted; however, the most likely contributors have been addressed and/or discussed, with clinicopathological correlations drawn. With the aim to revisit previously published alterations on different PGC-1α-deficient animals and to complete the phenotypic profiling of whole-body FL-PGC-1α-deficient mice specifically assessing non-motor behavioral alterations in light of age and sex, our findings extend the knowledge about the complex *in vivo* effect of PGC-1α dysfunction and add important notes to research in the field of PGC-1α in connection with neuropsychiatric disorders.

## Author Contributions

LS and MM performed the behavioral tests. LS and GK performed the histological analysis. LS, EC, GV, and DZ performed the HPLC analysis. LS and MM performed the statistical analyses. LS, DZ, LV, and PK contributed to the interpretation of the findings. LS and MM wrote the manuscript. LS constructed the figures and tables. All authors proofread and approved the manuscript.

## Conflict of Interest Statement

The authors declare that the research was conducted in the absence of any commercial or financial relationships that could be construed as a potential conflict of interest. The reviewer DG and handling Editor declared their shared affiliation at the time of the review.

## Abbreviations

AD: Alzheimer’s disease
BBB: blood–brain barrier
CNS: central nervous system
EPM: elevated plus maze
FLD: fluorescence detector
FL-PGC-1α: full-length PGC-1α
GR: glucocorticoid receptor
HD: Huntington’s disease
HPLC: high-performance liquid chromatography
IL: interleukin
KAT: kynurenine aminotransferase
KYNA: kynurenic acid
LDB: light-dark box
LEM6: ligand effect modulator 6
MND: motor neuron disease
NT-PGC-1α: N-terminal fragment of PGC-1α
OF: open-field
PD: Parkinson’s disease
PEPCK: phosphoenolpyruvate carboxykinase
PGC-1α: PPARγ coactivator-1alpha
PPARγ: peroxisome proliferator-activated receptor gamma
PV: parvalbumin
QUIN: quinolinic acid
TNFα: tumor necrosis factor alpha
TRP: tryptophan
TST: tail suspension test
