# Supplementary material for: Non-motor Behavioral Alterations of PGC-1α-Deficient Mice – A Peculiar Phenotype With Slight Male Preponderance and No Apparent Progression
Source: Front Behav Neurosci. 2018 Aug 27;12:180. doi: 10.3389/fnbeh.2018.00180 (PMC6119962; doi:10.3389/fnbeh.2018.00180)
Supplement: Supplementary file 1 [file Table_1.DOCX]

**Supplementary table 1.** Correlation of ancillary ambulatory parameters across different anxiety-related paradigms within FL-PGC-1α -/- mice

| **FL-PGC-1α -/- mice**  **(n = 26)** | *Total Distance*  *EPM* | *Ambulation Distance*  *OF* | *Light Velocity*  *LDB* |
| --- | --- | --- | --- |
| *Dark/Total Time*  *LDB* | R = 0.630 | R = 0.447 | R = 0.804 |
|  | p = 0.002 | p = 0.088 | p < 0.001 |
| *Total Distance*  *EPM* |  | R = 0.663 | R = 0.693 |
|  |  | p = 0.001 | p < 0.001 |
| *Ambulation Distance*  *OF* |  |  | R = 0.556 |
|  |  |  | p = 0.013 |

EPM, elevated plus maze; LDB, light-dark box; OF, open-field.
